# ITQ-54: a multi-dimensional extra-large pore zeolite with 20 × 14 × 12-ring channels†
†Electronic supplementary information (ESI) available: Synthesis, structure determination by RED, separation using the heavy liquid method, Rietveld refinement against PXRD of as-made and calcined samples, topology analysis, textural properties, ^29^Si and ^19^F solid state NMR, TG analysis and CIFs. CCDC 1021735. For ESI and crystallographic data in CIF or other electronic format see DOI: 10.1039/c4sc02577f



**DOI:** 10.1039/c4sc02577f

**Published:** 2014-10-08

**Authors:** Jiuxing Jiang, Yifeng Yun, Xiaodong Zou, Jose Luis Jorda, Avelino Corma

**Affiliations:** a Instituto de Tecnología Química (UPV-CSIC) , Universidad Politécnica de Valencia – Consejo Superior de Investigaciones Científicas , Av. de los Naranjos s/n , 46022 Valencia , Spain . Email: acorma@itq.upv.es; b Berzelii Center EXSELENT on Porous Materials and Inorganic and Structural Chemistry , Department of Materials and Environmental Chemistry , Stockholm University , SE-106 91 Stockholm , Sweden . Email: xzou@mmk.su.se

## Abstract

A multi-dimensional extra-large pore silicogermanate zeolite, named ITQ-54, has been synthesised by *in situ* decomposition of the *N*,*N*-dicyclohexylisoindolinium cation into the *N*-cyclohexylisoindolinium cation. Its structure was solved by 3D rotation electron diffraction (RED) from crystals of *ca.* 1 μm in size. The structure of ITQ-54 contains straight intersecting 20 × 14 × 12-ring channels along the three crystallographic axes and it is one of the few zeolites with extra-large channels in more than one direction. ITQ-54 has a framework density of 11.1 T atoms per 1000 Å^3^, which is one of the lowest among the known zeolites. ITQ-54 was obtained together with GeO_2_ as an impurity. A heavy liquid separation method was developed and successfully applied to remove this impurity from the zeolite. ITQ-54 is stable up to 600 °C and exhibits permanent porosity. The structure was further refined using powder X-ray diffraction (PXRD) data for both as-made and calcined samples.

## Abstract 2

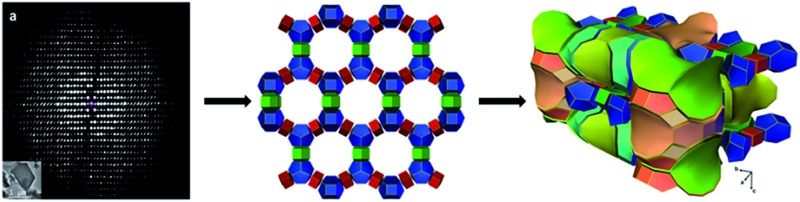
A silicogermanate zeolite with straight intersecting 20 × 14 × 12-ring channels has been synthesized and its structure has been solved using the rotation electron diffraction method.

## Introduction

Zeolites have attracted great interest because of their uniformly-sized pore structures in molecular dimensions, and they have wide industrial applications in catalysis, ion exchange, gas adsorption and separation.^1–3^ Zeolites with extra-large pore sizes (defined by >12 TO_4_ tetrahedra, T = Si, Al or Ge *etc.*) are desirable in order for large molecules to be able to diffuse through the channels. In particular, the presence of interconnected large and extra-large channels with different pore sizes and shapes in the same zeolite structure would be interesting, as it would allow both shape selectivity and fast diffusion of large molecules through the channels. Great efforts have been made to synthesize new zeolites,^4–6^ especially those with extra-large pore openings, multi-dimensional channels and low framework densities. However, extra-large pore zeolites are still very rare,^7,8^ especially zeolites with extra-large channels in more than one direction. The only examples are found in the gallophosphate cloverite and its aluminophosphate and silicogermanate analogues (-CLO, 20 × 20 × 20-ring),^9–11^ germanosilicates ITQ-37 (-ITV, gyroidal 30-ring channel),^12^ and ITQ-40 (16 × 15 × 15-ring).^13^ In addition, there are only three reported zeolite frameworks that have pore openings ≥20-rings, namely -CLO, -ITV and ITQ-43 (28 × 12 × 12-ring).^14^

Zeolites are often synthesised as polycrystalline powders. Powder X-ray diffraction (PXRD) can be used for structure solution, if the sample is pure and the structure is not too complicated. However, severe peak overlapping due to large unit cell and low symmetry precludes in most cases the structure elucidation by this technique. Electron diffraction (ED) has many advantages compared to X-ray diffraction for structure solution of nano- and micrometer-sized crystals. Crystals considered as powders by X-ray diffraction behave as single crystals by electron diffraction, which makes electron diffraction a promising technique for structural study.^15,16^ There is no ambiguity for unit cell and space group determination. Although ED data from zone-axis patterns has been used for structure solution of unknown zeolites,^17,18^ both the data collection and data processing are very demanding and require experts in electron crystallography. In addition, the ED data is incomplete and suffers severe dynamical effects.

Recently two methods for automated collection of three-dimensional electron diffraction data, automated diffraction tomography (ADT)^19^ and rotation electron diffraction (RED),^20–22^ have been developed. Both methods allow collection of almost complete 3D ED data sets from a nano- or micrometer-sized crystal starting with an arbitrary orientation. These methods not only make the structure solution from electron diffraction faster and more feasible, but also reduce dynamical effects. Several novel zeolite structures have been solved by the ADT and RED methods; for example a silicoaluminophosphate ITQ-51 with 16-ring channels,^23^ a germanosilicate PKU-16 with 11 × 11 × 12-ring channels,^24^ and a germanosilicate ITQ-43 with hierarchical meso-micropores.^14^ Here we report the silicogermanate ITQ-54 containing three-dimensional intersecting 20 × 14 × 12-ring channels. We show the power of the RED method in structure determination of unknown crystals from multiphasic samples.

## Results and discussion

The novel zeolite ITQ-54 was obtained using the *N*,*N*-dicyclohexylisoindolinium (DCHI) cation as the organic structure directing agent (OSDA). This cation was synthesised from 1,2-bis(bromomethyl)benzene and *N*,*N*-dicyclohexylamine (see ESI S2†). The zeolite was obtained after crystallisation at 200 °C for two days from a synthesis gel of composition:

0.62 SiO_2_:0.38 GeO_2_:0.29 OSDAOH:0.25 NH_4_F:1.5 H_2_O.

Elemental analysis shows a significant difference between the experimental C/N ratio measured in the sample (C/N = 14.4) and the theoretical value of the OSDA introduced in the synthesis gel (C/N = 20). This implies that the OSDA decomposed prior to its incorporation to the zeolite. Gas chromatography-mass spectrometry (GC-MS) and solid state ^13^C MAS-NMR of ITQ-54 show that the organic species occluded in the zeolite is the *N*-cyclohexylisoindolinium (CHI) cation (see Experimental section). The protonated CHI was generated *in situ* from DCHI during the synthesis, which seems to be the real OSDA to direct the synthesis of ITQ-54. However, synthesis attempts by using CHI as the sole template or as the co-template only produced even more GeO_2_ in the final product. When the Ge/Si ratio was decreased, the crystallinity of ITQ-54 also decreased, while the GeO_2_ content remained almost constant. So far, ITQ-54 can only be synthesised as an impure form by *in situ* decomposition of DCHI.

**Fig. 1:**
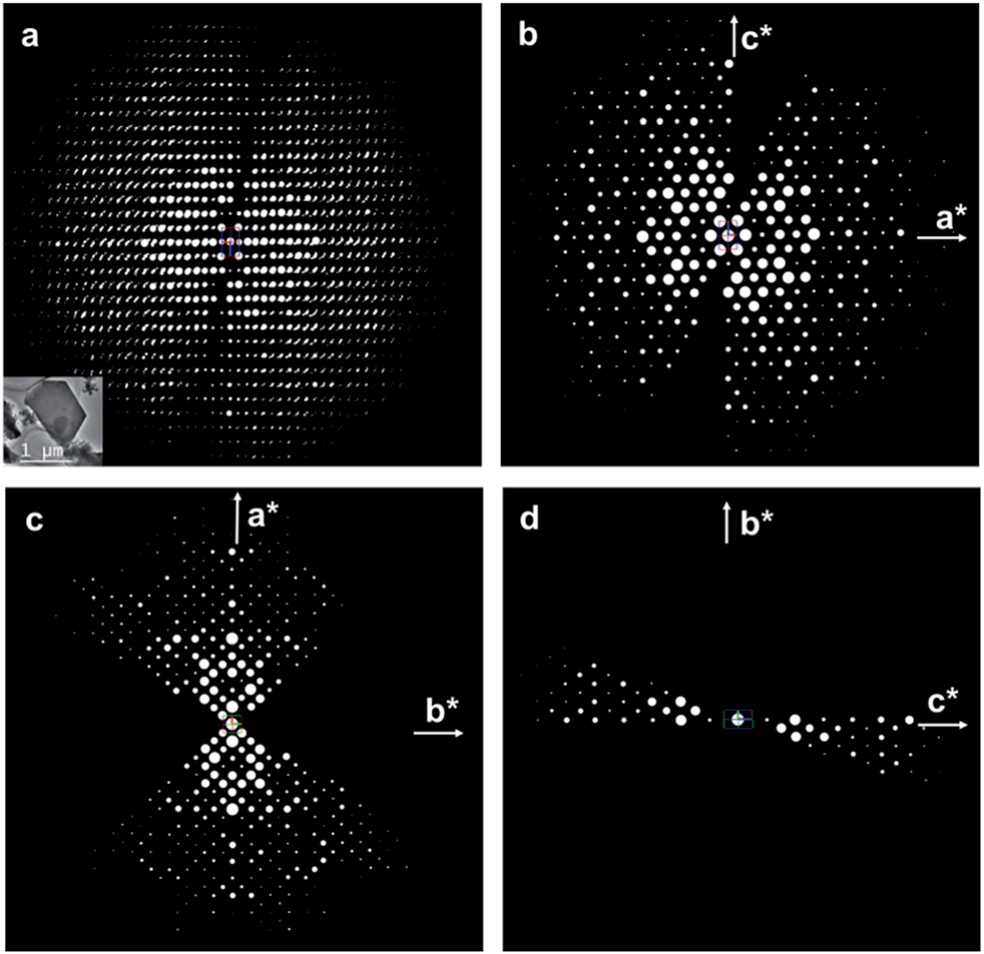
(a) 3D reciprocal lattice of ITQ-54 reconstructed from the RED data. The crystal from which the RED data was collected is shown in the insert. The intensity distribution in the *h*0*l* (b), *hk*0 (c) and 0*kl* (d) planes show 2*mm* symmetry, indicating that the crystal is orthorhombic. The reflection conditions can be deduced from the RED data as *hkl*: *h* + *k* + *l* = 2*n*, *hk*0: *h* + *k* = 2*n*, *h*0*l*: *h* + *l* = 2*n*, 0*kl*: *k* + *l* = 2*n*, *h*00: *h* = 2*n*, and 00*l*: *l* = 2*n*. The possible space groups are *Immm*, *Imm*2, *Im*2*m*, *I*222 and *I*2_1_2_1_2_1_.

The structure of ITQ-54 was determined using the RED method from an as-made sample containing GeO_2_ as the impurity (see Experimental section). 594 selected area electron diffraction (SAED) frames were collected from an ITQ-54 crystal of *ca.* 1 μm in size on a JEOL JEM2100 TEM using the software RED-Data Collection,^22^ with a tilt step of 0.20° and a tilt range of 109.31°. The SAED frames were combined to reconstruct the 3D reciprocal lattice of ITQ-54 using the software RED-Data Processing.^22^ The RED data shows that ITQ-54 has an *I*-centered lattice with unit cell parameters *a* = 26.59 Å, *b* = 24.93 Å, *c* = 15.94 Å, *α* = 89.91°, *β* = 90.83°, *γ* = 90.09°. The unit cell parameters were further refined against the PXRD data to be *a* = 26.9678(13) Å, *b* = 25.5772(13) Å, *c* = 16.2577(8) Å, *α* = *β* = *γ* = 90°. The reflection conditions were obtained from the 2D slices *h*0*l*, *hk*0 and 0*kl* cut from the 3D reciprocal lattice reconstructed from the RED data, as shown in Fig. 1. The possible space groups were deduced from the reflection conditions to be *Immm*, *Imm*2, *Im*2*m*, *I*222 and *I*2_1_2_1_2_1_. The structure of ITQ-54 was solved by direct methods using the highest symmetry, *Immm*. All nine symmetry-independent T-atoms (T = Si/Ge) and 21 symmetry-independent oxygen atoms in the framework were located directly. An additional peak was found in the center of a double 4-ring (D4R) in the difference Fourier map and assigned as fluorine anion.

All of the nine T atoms are tetrahedrally-coordinated to oxygen; eight are four-connected to other T-atoms and one is three-connected leaving a terminal hydroxyl group. The structure model was refined against the RED data and converged with *R*_1_ = 0.317 for 1913 symmetry-independent reflections. The RED data collection, crystal data and structure refinement details are given in Table S1.† The complete crystal structure determination of ITQ-54 by the RED method demonstrates its power for studying sub-micrometer sized crystals and samples containing impurities.

The comparison between ^29^Si cross polarisation (CP) MAS-NMR and ^29^Si Bloch decay (BD) MAS-NMR spectra (Fig. S12†) of the as-made sample shows the presence of Q^3^ species. This confirms the presence of terminal Si–OH groups as determined from the RED data. The ^19^F MAS-NMR spectrum (Fig. S13†) shows a single resonance band at –11.42 ppm, indicating that fluorine anions are trapped in the D4Rs, which also agrees with the structure model of ITQ-54 determined from the RED data.

The current RED data still suffers dynamical effects which gave a high *R*_1_ value in the structure refinement. In order to further confirm the structure model and obtain more accurate atomic positions, the structure needs to be refined against the PXRD data. Despite our efforts of synthesis optimisation, zeolite ITQ-54 was always obtained with a certain amount of GeO_2_. Thus we developed a separation method based on the different densities of the two compounds, using a heavy liquid to remove the GeO_2_ impurity as described below. In general, it is not unusual to obtain impurities such as amorphous, dense phases and other zeolitic phases during the synthesis of zeolites.^25–27^ Although impurities can be eliminated in most cases by optimizing the synthesis conditions, this approach sometimes fails. To the best of our knowledge, there are no reports about simple and general laboratory methods to eliminate impurities in zeolite samples. The only method to obtain a pure sample for property analyses is to pick out the crystals by hand under a microscope, based on the different crystal morphologies. However, this method is only applicable when crystals are both large and have well-defined shapes.^25,26^ Meanwhile, solid separation methods have already been used for decades in the mineral industry. Different minerals can be separated based on the difference of their densities using a heavy liquid media; phases with densities greater than the liquid will sink down, while those with densities less than the liquid will float to the surface. We found that a 1,1,2,2-tetrabromoethane (TBE)–ethanol solution (TBE, 2.967 g cm^–3^ Muthmann's solution) was suitable for the complete removal of GeO_2_ from the ITQ-54 sample, taking advantage of the large difference in the densities of ITQ-54 (2.18 g cm^–3^) and GeO_2_ (4.25 g cm^–3^). A pure ITQ-54 sample was successfully obtained by this approach. Details of the separation method are described in the Experimental section S5.†

Synchrotron PXRD data was collected from the as-made pure ITQ-54 sample (*λ* = 0.413686 Å). The structure model obtained by RED was refined against the synchrotron PXRD data using the program TOPAS Academic 4.1.^28^ The final refinement converged with *R*_wp_ = 0.0678, *R*_exp_ = 0.0535 and *R*_B_ = 0.0216, see Fig. S4 and Table S2.† All of the bond distances and angles in the refined structure of ITQ-54 are chemically reasonable. More details about the Rietveld refinement are described in the ESI.†

**Fig. 2:**
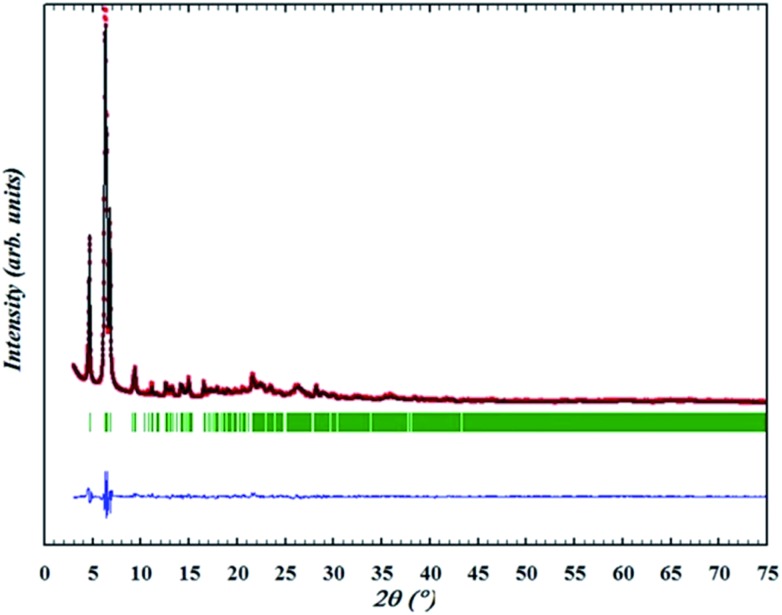
Rietveld refinement of the PXRD pattern of ITQ-54 upon calcination at 873 K (data collected at 303 K, Cu Kα radiation, *λ* = 1.5418 Å). Data points show the observed PXRD pattern; the line along these points is the calculated pattern, with the difference profile at the bottom. The vertical tick marks below the pattern give the positions of the Bragg reflections.

The OSDA could be completely removed by a slow stepwise calcination procedure while retaining the ITQ-54 framework, as described in the Experimental section S5.† Finally a calcined pure ITQ-54 sample was obtained, and its PXRD pattern was collected. Rietveld refinement was performed using the program FULLPROF,^29^ which converged with *R*_wp_ = 0.095, *R*_exp_ = 0.035, *R*_B_ = 0.038 and *R*_F_ = 0.041. The chemical formula of the calcined ITQ-54 is Si_0.51_Ge_0.49_O_1.97_(OH)_0.06_. The Rietveld plots are shown in Fig. 2 and crystallographic data is given in Table S3.†

**Fig. 3:**
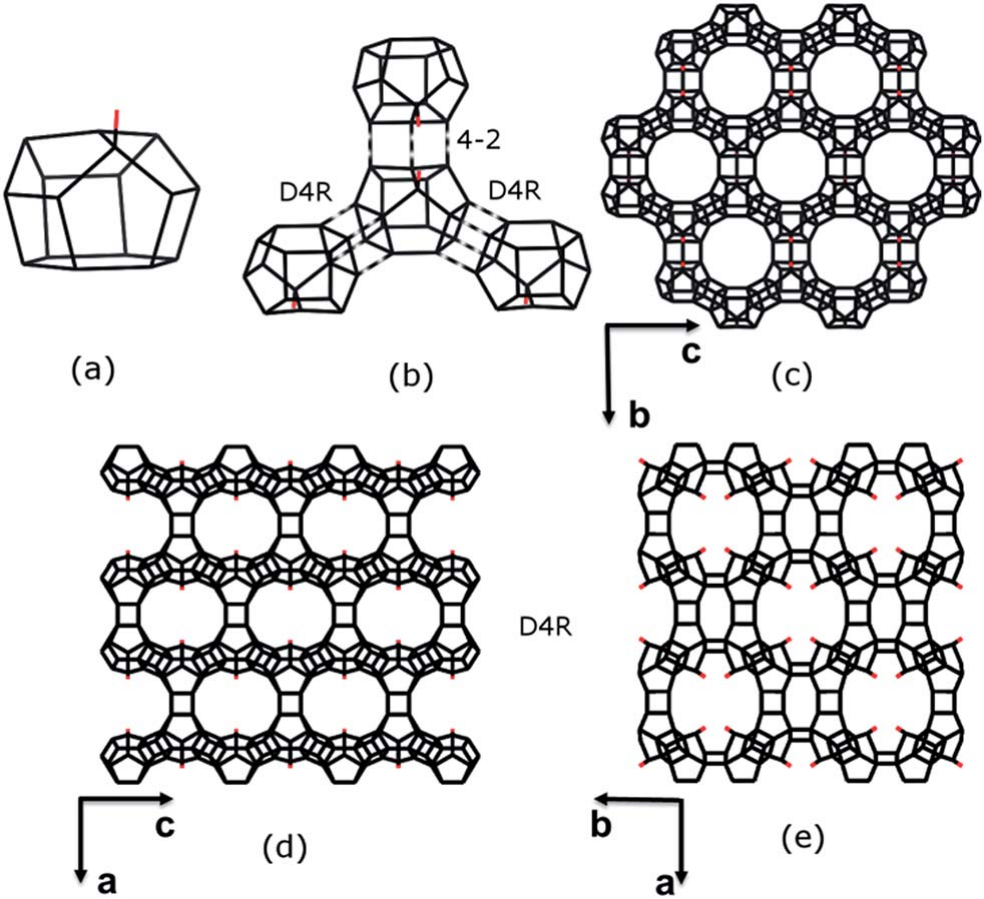
Construction of the framework structure of ITQ-54. (a) The new [4^3^5^6^6^1^] CBU. (b) Connectivity of the new CBU to the other three CBUs *via* D4R, D4R and 4–2 units, respectively, to form (c) a layer in the *bc*-plane with 12-ring pores. (d and e) The 12-ring layers connect to each other *via* D4Rs to form a 3D framework. 14-ring channels are created along the *b*-axis (d) and 20-ring channels are created along the *c*-axis (e). Only T–T connections and the terminal O atoms (in red) are shown.

The framework structure of ITQ-54 is built from a novel [4^3^5^6^6^1^] composite building unit (CBU) consisting of 16 TO_4_ tetrahedra (Fig. 3a). One of the TO_4_ tetrahedra in the [4^3^5^6^6^1^] CBU is three-connected, leaving a terminal –OH group. Each [4^3^5^6^6^1^] CBU connects to three neighbouring [4^3^5^6^6^1^] CBUs *via* their 4-rings to form two D4Rs and one 4–2 unit (Fig. 3b), defining a layer containing 12-rings in the *bc*-plane (Fig. 3c). Each layer is further connected to its closest neighbouring layers *via* additional D4Rs to form a 3D framework with tri-directional 20 × 14 × 12-ring channels (Fig. 3d and e). The free diameters are 10.3 Å × 5.8 Å for the 20-ring channels along the *c*-axis, 9.8 Å × 6.6 Å for the 14-ring channels along the *b*-axis and 7.2 Å × 6.8 Å for the 12-ring channels along the *a*-axis, as shown in Fig. S6.† ITQ-54 has a framework density of 11.1 T-atoms per 1000 Å^3^, which is one of the lowest among zeolites.^9,10,12,13,30–32^

**Fig. 4:**
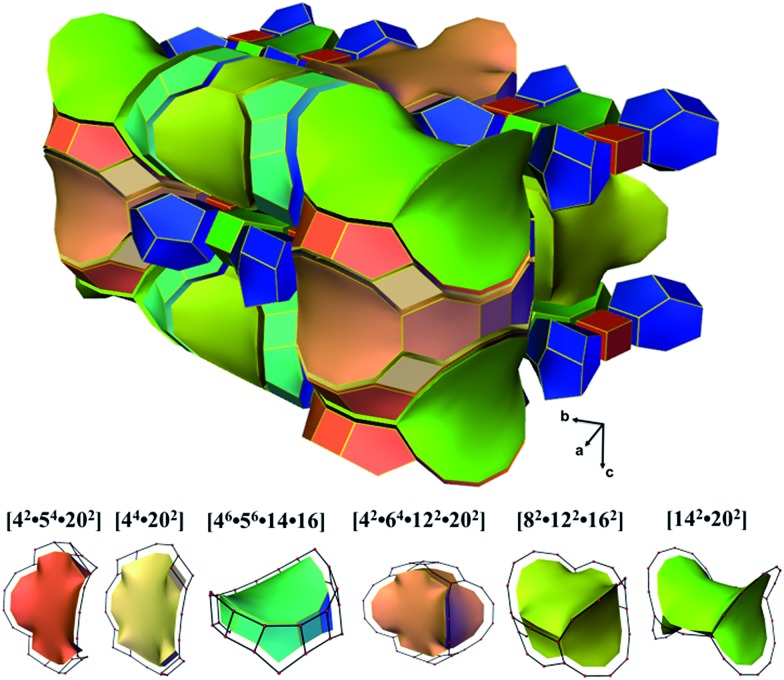
Illustration of the channel system and cavities in ITQ-54 by natural tiling. More descriptions are given in the ESI.†

ITQ-54 has a new zeolite topology, with intersecting extra-large channels (20- and 14-ring channels). The coordination sequences and vertex symbols are given in Table S5.† The channel system and cavities are better illustrated by natural tiling, where the intersection of the 20- and 14-ring channels as well as the 20-ring and 12-ring channels can be clearly seen by tracing the colours of the tiles (Fig. 4 and S7†). The framework can be described by a four-coordinated net crb with each node occupied by a [4^3^5^6^6^1^] CBU (Fig. S8†).

ITQ-54 is stable up to 600 °C and exhibits permanent porosity. Like most other zeolites with ≥20-rings, ITQ-54 contains germanium and the calcined sample is not stable in the presence of moisture. For practical applications, it is necessary to substitute Ge by Si and/or Al through direct or post-synthesis methods. These have been demonstrated in several germanosilicates.^33–35^ Similar procedures are promising to improve the thermal stability of ITQ-54, which is helpful for its use as a catalyst to process bulk molecules.

## Conclusions

In conclusion, a new zeolite ITQ-54 with extra-large pores has been synthesised. The zeolite synthesis was directed by *in situ* decomposition of the OSDA. Its structure was solved using the rotation electron diffraction (RED) method from a multiphasic sample. The structure of ITQ-54 contains straight intersecting 20 × 14 × 12-ring channels along the three crystallographic axes. It is one of the few zeolites with extra-large channels in more than one direction. The framework density of ITQ-54 also has one of the lowest framework densities among the known zeolites. The multi-dimensional extra-large channels in ITQ-54 can facilitate the diffusion of large molecules through the channels, which is especially interesting for applications in separation and catalysis. The hydroxyl groups pointing towards the channels can be used for further functionalisation of the material. We have successfully applied a separation method based on the use of a heavy liquid to remove GeO_2_ and obtain pure ITQ-54 samples. We show the power of the RED method in the structure solution of submicrometer-sized crystals, and in discovering new structures in multi-phasic samples. This is important for the targeting of interesting materials so that the synthesis conditions can be optimised according to the structures.

## Experimental sections

### Synthesis

In a typical synthesis of zeolite ITQ-54, Ludox (SiO_2_, AS-40, 40% Sigma-Aldrich), OSDA (8–16% in water solution), GeO_2_ (99.998%, Sigma-Aldrich) and NH_4_F (99.99% Aldrich, 10 wt% in water solution), were introduced into 3 ml Teflon vials and stirred for ∼3 h under infrared lamp heating. The air temperature around the synthesis gel was controlled to be within 80–90 °C using a thermometer, and the intensity of infrared light was adjusted to ensure a proper decomposition of the OSDA and evaporation of water until the desired water content was achieved. Then, the Teflon vials were inserted into the 3 × 5 multiautoclave and the crystallisation was carried out at 200 °C for two days in static conditions. Crystalline products were washed with abundant quantities of water and acetone prior to drying at 100 °C. The samples were characterised by powder X-ray diffraction; all of the as-made samples contained some GeO_2_ impurity which could not be avoided regardless of synthesis optimisation. The initial sample used for structure elucidation by electron crystallography was synthesised in a gel composition of 0.62SiO_2_:0.38GeO_2_:0.29OSDAOH:0.25NH_4_F:1.5H_2_O. The synthesis was optimised into a gel composition of 0.68SiO_2_:0.32GeO_2_:0.25OSDAOH:0.23NH_4_F:1.1H_2_O and the resulting sample was used for the study of GeO_2_ removal and further characterisations.

Compared with previous silicogermanate zeolite syntheses, the synthesis of ITQ-54 needs a relative high temperature (80–90 °C) during the gel preparation to induce the proper decomposition of the OSDA. If the heating temperature is not high enough, the product either contains BEC impurity or transforms completely to the BEC zeolite. If the heating temperature is too high, the product becomes trigonal GeO_2_.

### Analysis of occluded organic species in ITQ-54 by GC-MS and ^13^C NMR

In order to know which organic species are occluded, 2 g of as-made ITQ-54 were dissolved with 37% HCl, and the organic species was extracted with 100 ml of chloroform. The organic layer was first washed by saturated K_2_CO_3_ and anhydrous MgSO_4_ solutions, and then analysed by gas chromatography-mass spectrometry (GC-MS). One major peak (73.9% estimated by area integration) and several minor peaks (total 26.1%) were observed in the gas chromatography spectra. The main peaks can be recognised as *N*,*N*-cyclohexylisoindolinium (CHI) by comparing the GC-MS spectra with synthetic CHI (Fig. S9†). The solid state ^13^C MAS-NMR of ITQ-54 and the liquid ^13^C NMR of the protonated CHI are in good agreement (Fig. S10†), as well as the C/N ratio (experimental = 14.4, theoretical = 14).

### Structure determination by RED

Powders of the as-made ITQ-54 were crushed, dispersed in absolute ethanol and treated by sonication for 2 minutes. Then, a droplet of the suspension was transferred onto a copper grid. The TEM sample was observed on a JEOL JEM-2100 microscope operated at 200 kV using a single-tilt tomography sample holder. The RED data sets were collected using the software package RED-data collection.^22^ 594 SAED frames were collected covering a tilt range of 109.31°. Energy-dispersive spectroscopy (EDS) was carried out on the same crystals after the RED data collection to determine the Si/Ge ratio. The SAED frames were combined to reconstruct the 3D reciprocal lattice using the software RED-data processing,^22^ which performed shift correction, peak search, unit cell determination, indexation of the reflections and intensity extraction. A list of *hkl* intensity was extracted from the RED data and used for the structure solution by direct methods, followed by the structure refinement. The intensities *I*(*hkl*) obtained from RED were directly used as |*F*(*hkl*)|^2^ for structure determination, *i.e.* using kinematical approximation. Final structure refinement was performed using the SHELXL-97 program by minimising the sum of squared deviations of *F* ^2^ using a full-matrix technique. All of the T (T = Si/Ge), O and F atoms were refined isotropically.

### Separation of ITQ-54 and GeO_2_

The separation was performed by the heavy liquid method based on the different densities of the zeolite and the GeO_2_ impurity. A TBE–ethanol solution (density 2.21 g cm^–3^) was chosen as a working medium. Before the separation, the sample was dispersed in ethanol to break the aggregation of both species (see ESI†). After a first separation in a 2.21 g cm^–3^ (87.1%) TBE–ethanol solution, the top layer (2.21 g) and bottom layer (0.21 g) were collected separately. The GeO_2_ content in the top layer decreased from 8.7% to 1.5%, based on density contribution. The separation procedure was then repeated twice, with the GeO_2_ content decreased to 0.97% and <0.5%, respectively. In fact, in the PXRD pattern of the last sample, no diffraction peaks from GeO_2_ could be observed.

### Powder X-ray diffraction

Synchrotron PXRD data was collected from the as-made pure ITQ-54 sample on the beamline 11-BM at the Advanced Photon Source, Argonne National Laboratory (*λ* = 0.413686 Å). PXRD data of the calcined pure ITQ-54 was collected on a PANalytical X'Pert PRO diffractometer from a sample obtained through direct *in situ* calcination of the as-made pure ITQ-54 sample in an Anton Parr XRK-900 chamber, at a heating rate of 3 °C min^–1^. The sample was kept at constant temperatures for 2 hours at 100, 150, 200, 250 and 300 °C, and for 4 hours at 350, 400, 450, 500, 550 and 600 °C. The PXRD pattern was collected after the sample being cooled down to room temperature.

### Topology analysis

The nets of ITQ-54 were computed using TOPOS.^36,37^ The visualisation of nets and tiling was generated using 3dt.^38^

## References

[cit1] Davis M. E. (2002). Nature.

[cit2] Atienzar P., Díaz-Cabañas M. J., Moliner M., Peris E., Corma A., García H. (2007). Chem.–Eur. J..

[cit3] Corma A. (2003). J. Catal..

[cit4] Wang Z., Yu J., Xu R. (2012). Chem. Soc. Rev..

[cit5] Li Y., Yu J., Xu R. (2013). Angew. Chem., Int. Ed..

[cit6] Meng X., Xiao F.-S. (2014). Chem. Rev..

[cit7] Baerlocher C. and McCusker L., 2014, Database of Zeolite Structures: http://www.iza-structure.org/databases/, (accessed August 20, 2014).

[cit8] Jiang J., Yu J., Corma A. (2010). Angew. Chem., Int. Ed..

[cit9] Estermann M., McCusker L. B., Baerlocher C., Merrouche A., Kessler H. (1991). Nature.

[cit10] Su J., Wang Y., Lin J., Liang J., Sun J., Zou X. (2013). Dalton Trans..

[cit11] Wei Y., Tian Z., Gies H., Xu R., Ma H., Pei R., Zhang W., Xu Y., Wang L., Li K., Wang B., Wen G., Lin L. (2010). Angew. Chem., Int. Ed..

[cit12] Sun J., Bonneau C., Cantín Á., Corma A., Díaz-Cabañas M. J., Moliner M., Zhang D., Li M., Zou X. (2009). Nature.

[cit13] Corma A., Díaz-Cabañas M. J., Jiang J., Afeworki M., Dorset D. L., Soled S. L., Strohmaier K. G. (2010). Proc. Natl. Acad. Sci. U. S. A..

[cit14] Jiang J., Jorda J. L., Yu J., Baumes L. A., Mugnaioli E., Diaz-Cabanas M. J., Kolb U., Corma A. (2011). Science.

[cit15] McCusker L., Baerlocher C. (2013). Z. Kristallogr..

[cit16] Willhammar T., Yun Y., Zou X. (2014). Adv. Funct. Mater..

[cit17] Dorset D. L. (2003). Z. Kristallogr..

[cit18] Wagner P., Terasaki O., Ritsch S., Nery J. G., Zones S. I., Davis M. E., Hiraga K. (1999). J. Phys. Chem. B.

[cit19] Kolb U., Gorelik T., Kübel C., Otten M. T., Hubert D. (2007). Ultramicroscopy.

[cit20] Zhang D., Oleynikov P., Hovmöller S., Zou X. (2010). Z. Kristallogr..

[cit21] Zou X., Hovmoller S. and Oleynikov P., Electron Crystallography: Electron Microscopy and Electron Diffraction, Oxford University Press, 2011.

[cit22] Wan W., Sun J., Su J., Hovmoller S., Zou X. (2013). J. Appl. Crystallogr..

[cit23] Martinez-Franco R., Moliner M., Yun Y., Sun J., Wan W., Zou X., Corma A. (2013). Proc. Natl. Acad. Sci. U. S. A..

[cit24] Hua W., Chen H., Yu Z.-B., Zou X., Lin J., Sun J. (2014). Angew. Chem., Int. Ed..

[cit25] Conradsson T., Dadachov M. S., Zou X. D. (2000). Microporous Mesoporous Mater..

[cit26] Liu Z., Song X., Li J., Li Y., Yu J., Xu R. (2012). Inorg. Chem..

[cit27] Tang L., Shi L., Bonneau C., Sun J., Yue H., Ojuva A., Lee B.-L., Kritikos M., Bell R. G., Bacsik Z., Mink J., Zou X. (2008). Nat. Mater..

[cit28] Young R. A., The Rietveld Method, Oxford University Press, 1995.

[cit29] Rodríguez-Carvajal J. (1993). Phys. Rev. B: Condens. Matter Mater. Phys..

[cit30] Zheng N., Bu X., Wang B., Feng P. (2002). Science.

[cit31] Xu Y., Li Y., Han Y., Song X., Yu J. (2013). Angew. Chem., Int. Ed..

[cit32] Han Y., Li Y., Yu J., Xu R. (2011). Angew. Chem., Int. Ed..

[cit33] Gao F., Jaber M., Bozhilov K., Vicente A., Fernandez C., Valtchev V. (2009). J. Am. Chem. Soc..

[cit34] Xu H., Jiang J., Yang B., Zhang L., He M., Wu P. (2014). Angew. Chem., Int. Ed..

[cit35] Burel L., Kasian N., Tuel A. (2014). Angew. Chem., Int. Ed..

[cit36] Blatov V. A., Shevchenko A. P., Proserpio D. M. (2014). Cryst. Growth Des..

[cit37] Blatov V. A., Shevchenko A. P., Serezhkin V. N. (2000). J. Appl. Crystallogr..

[cit38] Delgado-Friedrichs O. (2003). Theor. Comput. Sci..

