# Supplementary material for: ITQ-54: a multi-dimensional extra-large pore zeolite with 20 × 14 × 12-ring channels
Source: Chem Sci. 2014 Oct 8;6(1):480–5. doi: 10.1039/c4sc02577f (PMC5811170; doi:10.1039/c4sc02577f)
Supplement: Supplementary file 1 [file SC-006-C4SC02577F-s001.pdf]

Electronic Supplementary Material (ESI) for Chemical Science.
This journal is © The Royal Society of Chemistry 2014

Supporting information

ITQ-54: a multi-dimensional extra-large pore zeolite with 20 × 14 × 12-ring channels

Jiuxing Jiang, [‡ a] Yifeng Yun, [‡ b], Xiaodong Zou,[*b] Jose Luis Jorda,[a] and Avelino Corma[*a]

[a] DepartmeInstituto de Tecnología Química (UPV-CSIC), Universidad Politécnica de Valencia - Consejo Superior de Investigaciones Científicas, Av. de los Naranjos s/n, 46022 Valencia, Spain

[b] Berzelii Center EXSELENT on Porous Materials and Inorganic and Structural Chemistry, Department of Materials and Environmental Chemistry, Stockholm University, SE-106 91 Stockholm, Sweden

E-mail: acorma@itq.upv.es
E-mail: xzou@mmk.su.se

[‡] These authors contributed equally to this work

Contents

S1. Characterisation and methods
S2. Synthesis of the organic structure directing agents (OSDAs)
S3. Removal of GeO_2 impurity
S4. Structural determination of the as-made zeolite ITQ-54 using rotation electron diffraction (RED) data
S5. Structural refinement of the as-made and calcined zeolite ITQ-54 using powder X-ray diffraction (PXRD) data
S6. Structure and topology analysis of ITQ-54
S7. Characterisation of OSDA
S8. Textural properties of ITQ-54
S9. ^{29}Si and ^{19}F solid state NMR
S10. Thermogravimetrical analysis

S1. Characterisation and methods

The centrifugation was performed in Beckman Coulter Avanti J-26XP, with a rotor JA30.50, and using 40ml centrifuge tubes.

Laboratory X-ray powder diffraction data of the as-made and calcined ITQ-54 samples were collected in a Anton Paar XRK-900 chamber attached to a PANalytical X-Pert Pro diffractometer using Bragg-Brentano geometry, with Cu Kα radiation (45 kV, 40 mA) and an X'Celerator detector.

A 30 mg sample for ICP (Inductively Coupled Plasma) analysis was dissolved in a mixed acid solution 1ml HF (40%, Merck Suprapur®), 1ml HNO_3 (65%, Merck, Emsure®), 3ml HCl (30%, Merck Suprapur®) at room temperature 24h and then diluted to 60g with distillated water and sent to VARIAN715-ES ICP-PLASMA.

Gas chromatography data were obtained on Agilent 6890N with GC column HP-5MS. The temperature program was: 50ºC 2 min, 30 ºC/min to 280ºC 15 min. Gas flow: 1.2 ml/min He. Split Injector 1:60, 250 ºC.

Mass spectra were obtained with Agilent 5973N mass selective detector.

Thermogravimetric analyses were performed on a METTLER TOLEDO TGA/SDATA851e, from room temperature to 800ºC with a heating rate of 10ºC/min.

^{1}H and ^{13}C liquid-NMR data were measured for the organocations in solution ($CDCl_3$ as solvent) on a Bruker Avance300 spectrometer. The solid-state MAS-NMR spectra were recorded at room temperature on a Bruker AV400 spectrometer. ^{1}H to ^{13}C CP (cross-polarised) MAS-NMR spectra were recorded with proton decoupling, with 90º pulse length for ^{1}H of 4.5 ms, a contact time of 3 ms and recycle delay of 3s. ^{29}Si BD (bloch decay) MAS-NMR spectra were measured using pulses of 4 ms corresponding to a flip angle of π/3 radians and recycle delay of 240s. The ^{1}H to ^{29}Si CP MAS-NMR spectra were recorded using a 90º pulse length for ^{1}H of 4.5 ms, and a contact time of 1 ms and recycle delay of 3s. The ^{13}C and ^{29}Si spectra were recorded using a BL-7 probe with 7 mm diameter zirconia rotors spinning at 5 kHz. ^{19}F MAS-NMR spectra were recorded at 25 kHz spinning speed in a BL2.5 probe using 2.5 mm diameter rotor with a 90º pulse length of 5 ms and a recycle delay of 60s. The ^{13}C, ^{29}Si and ^{19}F spectra were referred to adamantane (CH_2 signal at 38.3 ppm), TMS (0 ppm), and $CFCl_3$ (0 ppm) respectively.

The sample for textural analysis was calcined at 540ºC (with a heating rate of 2ºC/min from room temperature) for 6h in a dry air atmosphere. During the cooling to room temperature, the gas flow was substituted by N_2 until the sample was sealed in a gas adsorption tube to be degassed. The N_2 adsorption curve was measured on a Micromeritics ASAP 2420, degassing at 400ºC. The Argon adsorption was measured on a Micromeritics ASAP 2020, after degassing also at 400ºC.

S2. Synthesis of the organic structure directing agents (OSDAs)

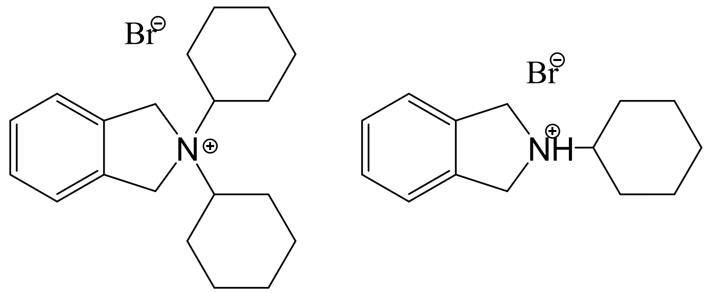

N,N-dicyclohexylisoindolinium bromide (a) DCHI C/N=20

2-cyclohexylisoindolium bromide (b) CHI C/N=14

Figure S1. OSDAs used in the synthesis of ITQ-54. (a) DCHI, the organic structure directing agent introduced in synthesis; (b) CHI, the actual guest molecule formed by *in situ* decomposition of DCHI.

Synthesis of the OSDA N,N-dicyclohexylisoindolinium hydroxide (DCHI)

The synthesis procedure was adapted from a literature method.[39]

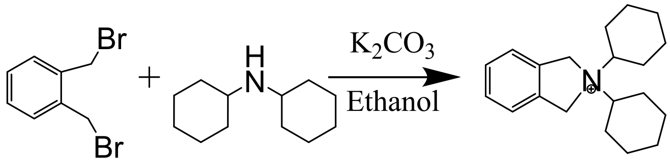

(a) (b)

In a typical synthesis, 50 g 1,2-bis(bromomethyl)benzene (0.189 mol, 97%, Sigma-Aldrich), 34.34 g N,N-dicyclohexylamine (0.189 mol, 99%, Sigma-Aldrich), 26.18 g K_2CO_3 (99.99%, Sigma-Aldrich) and 200 ml ethanol were mixed in a 1 l flask, and heated to reflux for 2 days. Then the solution, with a white precipitate, was evaporated to dryness in a rotavapor to remove all the remaining solvent. The product was dissolved in 200ml chloroform, precipitated K_2CO_3 was filtered off, and the chloroform was again evaporated, resulting in dark brown oil. 100ml water and 100ml diethyl ether were added, and the mixture was shaken until a large amount of white precipitate was formed. The solid was recovered by filtration, and washed with diethyl ether. To recover the product dissolved in water, this phase was separated from the diethyl ether evaporated to dryness to recover the solid, which was combined with the previous product (yield around 75%). The product was exchanged to the hydroxide form with an anionic exchange Amberlite IRN-78 resin in batch overnight.

Synthesis of the OSDA N,N-cyclohexylisoindolinium (CHI)

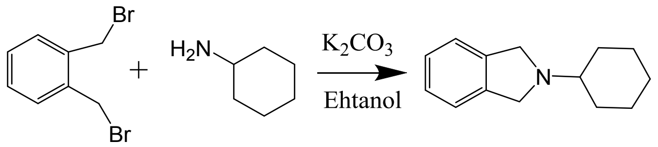

In a typical synthesis, 10 g 1,2-bis(bromomethyl)benzene (0.038 mol, 97%, Sigma-Aldrich), 6.87 g N-cyclohexylamine (0.038 mol, 99%,Sigma-Aldrich), 5.24 g K_2CO_3 (99.99%, Sigma-Aldrich) and 50 ml ethanol were mixed in a 500 ml flask and heated to reflux for 2 days. Then the solution, with a white precipitate, was evaporated to dryness in a rotavapor to remove all the remaining solvent. The product was extracted with 200 ml chloroform, precipitated K_2CO_3 was removed by filtration and the chloroform was evaporated, resulting in dark brown oil. After dissolving the oil in diethyl ether, dry HCl gas was bubbled in the solution until no precipitate was formed. The white precipitate was recovered by filtration and dried, resulting in 6.5 g of product (yield around 60%).

S3. Removal of GeO_2 impurity

Under the optical microscope, the sample particles are neither monodispersed nor well shaped. It implies that the zeolite particles are physically combined with GeO_2 particles. Thus, before the separation, the sample was dispersed in ethanol to break the aggregation of both species.

2.43 g of the as-made sample were finely grinded and dispersed into 250ml ethanol, and treated in an ultrasonic water bath for 30min. The precipitate was decanted and the suspension was centrifuged to separate the dispersed solid from the ethanol. The recovered ethanol was recycled to disperse again the previous precipitate. This process was repeated until all the sample was dispersed in ethanol. Finally, all the dispersed samples were mixed.

The separation was based on the different densities of the zeolite and the GeO_2 impurity.

Through the density analysis, the gross density of the as-made sample is 2.36 g/cm^3, while the hexagonal GeO_2 has a density of 4.25 g/cm^3. So any liquid with a density in-between those two values could be used to separate the phases. In this work, we used a non-water based heavy liquid due to the hydrophobic nature of the sample. Thus, the classic TBE was chosen as a working medium. Considering that some ethanol can remain in the dispersed sample, ethanol was selected as co-solvent to avoid a sample drying process that could cause particle re-agglomeration. To control the density of the TBE-ethanol mixture, a density vs percentage curve at ambient temperature (25ºC) was drawn and fitted, as seen in Figure S2.

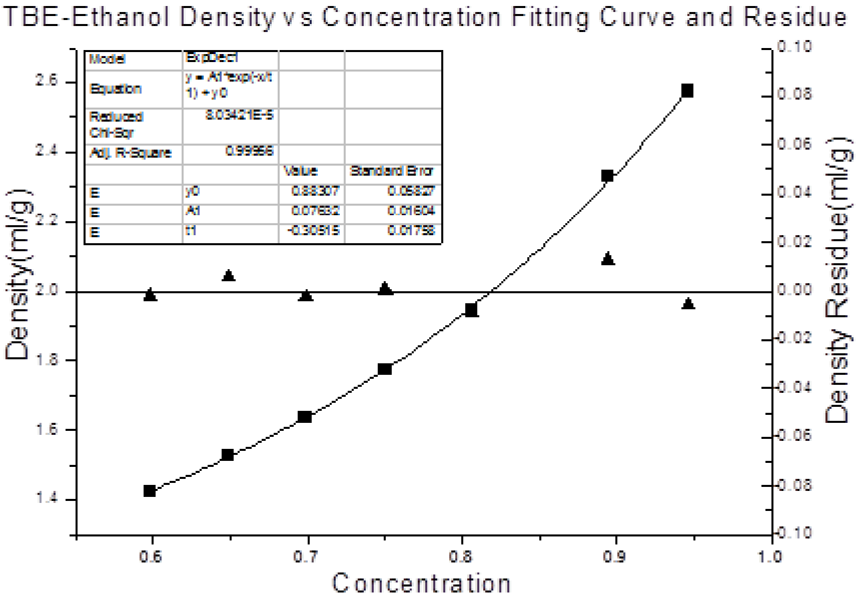

Figure S2. TBE-ethanol density vs TBE concentration fitting curve and residue.

According to the curve, the density of the solution can be well controlled by changing the TBE/ethanol ratio. Except otherwise noticed, all the density values of the solutions in this work were calculated based on its concentration.

Before the first attempt of separation, the sample was dispersed in a liquid mixture with a density of 2.10 g/cm^3. After addition of a calculated amount of TBE, the density reached 2.18 g/cm^3 and a small portion of sample particles started to float in the solution, indicating that the real zeolite density is around this value. So, the GeO_2 content of any mixture of ITQ-54 (2.18 g/cm^3) and GeO_2 (4.25 g/cm^3) could be roughly estimated from its experimental density. For the as-made sample, the gross density is 2.34 g/cm^3 (determined by the density bottle method), that corresponds to an 8.7% GeO_2 content. After a first separation in a 2.21 g/cm^3 (87.1%) TBE-ethanol solution, the top layer (2.21 g) and bottom layer (0.21 g) were collected separately. PXRD (Figure S3) shows that the intensities of the two diffraction peaks of GeO_2, at $2\theta = 20.60^\circ$ and 26.11°, of the top layer decrease while those in the bottom layer increase. The GeO_2 content in the top layer decreased from 8.7% to 1.5%, based on density contribution.

The separation procedure was then repeated twice, with the GeO_2 content decreased to 0.97% and <0.5%, respectively. In fact, in the PXRD pattern of the last sample, no diffraction peaks from GeO_2 could be observed.

Thus, we can state that the GeO_2 impurity can be successfully removed by the heavy liquid method.

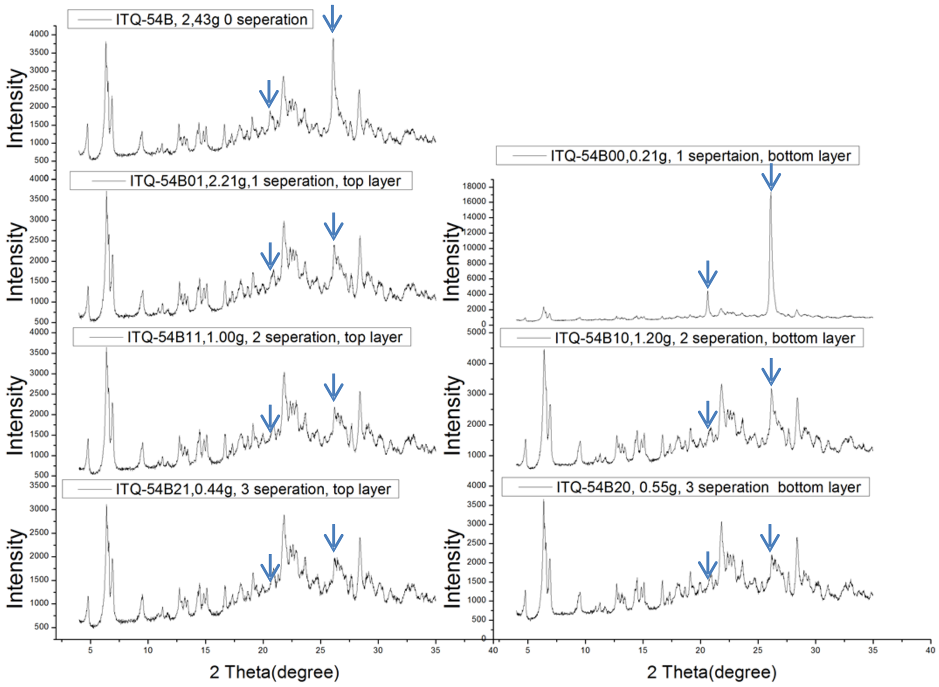

Figure S3. PXRD patterns of samples before separations and two layers components after separations.

We would like to mention that the classical heavy liquids like 1,1,2,2-tetrabromoethane (TBE, 2.967g/cm^3 Muthmanns solution), potassium tetraiodomercurate(II) (3,196 g/cm^3, Thoulets solution), bromoform and diiodomethane used in mineralogy are toxic. To avoid toxic chemicals there are alternative water based, non-toxic heavy liquids like sodium polytungstate (SPT) and lithium heteropolytungstates (LST). With these heavy liquids densities up to 4.2 g/cm can be adjusted.[40-42]

S4. Structural determination of the as-made zeolite ITQ-54 using rotation electron diffraction (RED) data

The RED data collection and data processing are described in the Experimental Section.

The as-made samples studied by TEM were not pure and contained plate-like ITQ-54 crystals as the major phase, and germanium dioxide and amorphous materials as impurities. The phase identification was performed using the RED method. The lattice type and unit cell parameters for both ITQ-54 and germanium dioxides were determined from the RED data. The structure solution using the RED data was carried out by direct methods using the SHELXS-97 program with the space group *Immm*.

Nine symmetry-independent T-atoms (T = Si, Ge) and 21 symmetry-independent oxygen atoms in the framework were located directly by direct methods. Another peak was found in the center of a

double 4-ring (D4R), which is assigned as fluorine anion, which was also confirmed by the solid state ^{19}F-MAS-NMR (Figure S12). The structure model was refined against the RED data and converged with R1 = 0.317 for 1913 independent reflections. The structure has a 3D open-framework and all the T-atoms are tetrahedrally-coordinated to oxygen atoms. All the bond distances and bond angles are reasonable. The high R1 value could be due to the contribution of the OSDAs in the pores, dynamical effects and the lack of absorption correction. The beam damage also affected the intensities.

Table S1. 3D-RED data collection, crystal data and structure refinement details of ITQ-54 (resolution cut to 1.0 Å)

	ITQ-54
Tilt range	+ 62.79° to – 46.52°
Tilt step	0.20°
No. of ED frames	594
Exposure time/frame	1.0 s
Space group	*Immm*
a / Å	26.59
b / Å	24.93
c / Å	15.94
α / °	89.91
β / °	90.83
γ / °	90.09
Completeness	62.7%
R_{int}	0.325
No. of measured reflections	20809
No. of independent reflections	1913
h	-26 ≤ h ≤ 26
k	-19 ≤ k ≤ 19
l	-15 ≤ l ≤ 15
*R*1	0.317
No. of parameters	112

S5. Structural refinement of the as-made and calcined zeolite ITQ-54 using powder X-ray diffraction (PXRD) data

Rietveld refinement of the as-made zeolite ITQ-54

Synchrotron PXRD data of as-made ITQ-54 after removing the GeO_2 was collected through the mail-in system of beamline 11-BM at the Advanced Photon Source, Argonne National Laboratory, USA. The wavelength was 0.413686 Å. Rietveld refinement was performed on the structure model obtained by RED using TOPAS Academic 4.1[28]. An additional F atom was added in the double 4-ring of ITQ-54 before the refinement. Soft restraints for the T-O bond distances were applied for ITQ-54. The OSDA in ITQ-54 could not be located. Instead, 15 carbon atoms were added at random positions inside the pores to compensate for the electron density of the OSDAs and subsequently refined. The refinement converged with $R_F = 0.0216$, $R_{wp} = 0.0678$ and $R_{exp} = 0.0535$, as shown in Figure S4 and Table S2. All the bond distances and bond angles are chemically reasonable in the refined structure of ITQ-54. Details of the 3D-RED data collection, crystal data and structure refinement of ITQ-54 are given in Table S1 and Table S2**.**

Table S2. Crystallographic data for Rietveld refinement of as-made ITQ-54

Chemical formula (per unit cell)	$\|(C_{14}NH_{20})_8\|[Si_{66.17}Ge_{61.83}O_{252}(OH)_8F_8(HF)_4]$
Chemical formula ($Z = 128$)	$\|(C_{14}NH_{20})_{0.06}\|[Si_{0.52}Ge_{0.48}O_{1.97}(OH)_{0.06}F_{0.06}(HF)_{0.03}]$
Formula weight (per unit cell)	12368 g/mol
a	26.9678(13)Å
b	25.57725785(13) Å
c	16.2577(8)Å
V	11213.94(94) Å^3
Space group	*Immm*
Synchrotron wavelength	0.413686 Å
No. of reflections	2410
No. of parameters	189
No. of restraints	36 for T-O, 64 for O-T-O and one for the total Ge/Si ratio
R_p	0.0557
R_{wp}	0.0678
R_{exp}	0.0535
GOF	1.267

R_F	0.0216

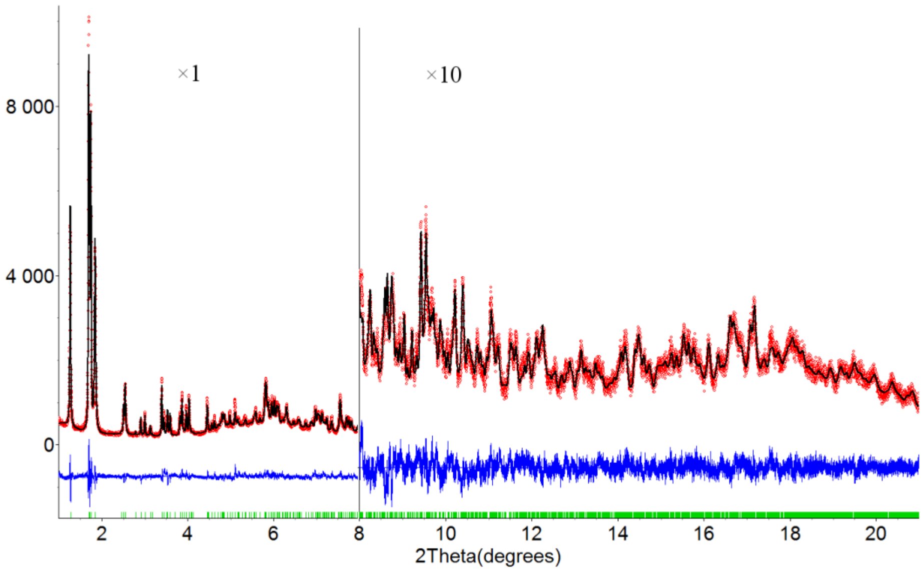

Figure S4. Observed (red data points) and calculated (black line) PXRD profiles, as well as the difference between observed and calculated profile (blue), for the Rietveld refinement of the as-made ITQ-54 (λ = 0.413686 Å).

Rietveld refinement of the calcined zeolite ITQ-54

In situ direct calcination of the sample was first attempted in an Anton Parr XRK-900 chamber attached to a PANalytical X'Pert PRO diffractometer, using a heating rate of 3 ºC/min up to 650 ºC. However, such process resulted in a clear loss of crystallinity of the material even at 550 ºC, as shown in Figure S5. Only the GeO_2 peaks were observable after calcination.

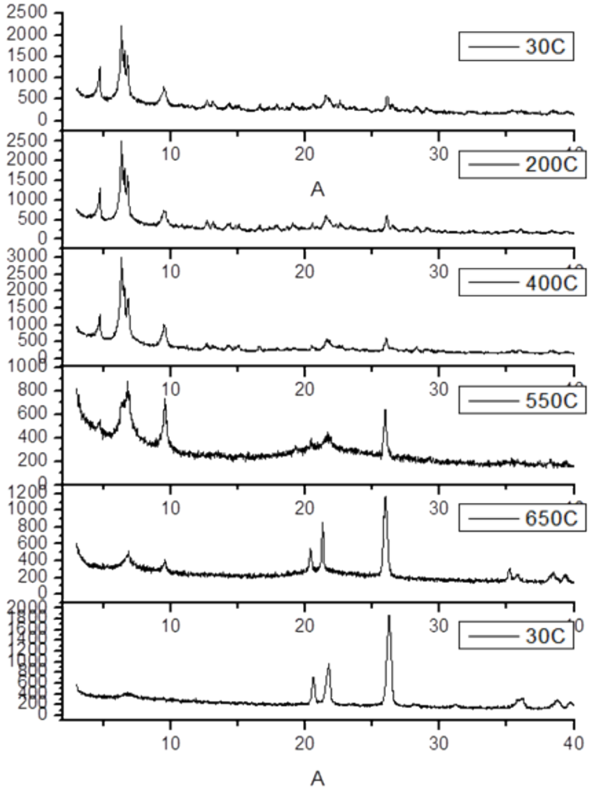

Figure S5. Temperature dependent PXRD patterns of ITQ-54.

After obtaining a new, GeO_2-free sample of ITQ-54, *in situ* calcination was repeated using a different approach. In this case the calcination process was much slower, keeping the sample at constant temperature for 2 hours at 100, 150, 200, 250 and 300 ºC, and for 4 hours at 350, 400, 450, 500, 550 and 600 ºC. After being cooled down to room temperature, the calcined ITQ-54 showed highly crystallinity, as seen by its PXRD pattern. The Rietveld refinement of the calcined ITQ-54 material was performed using the program FULLPROF,[28] and converged with R_{wp} = 0.095, R_{exp} = 0.035, R_B = 0.038, and R_F = 0.041, as shown in Figure 2 and Tables S3-4. In this case, the refinement confirmed that the contribution of GeO_2 is negligible.

Table S3: Crystallographic data for Rietveld refinement of calcined ITQ-54

PXRD data collection

Difractometer: PANalytical X'Pert PRO

Detector: PANalytical X'Celerator

Geometry: Bragg-Bretano geometry

X-ray radiation: Cu Kα (λ_1 = 1.5406 Å, λ_2 = 1.5444 Å, I_2/I_1 = 0.5)

Divergence slit: fixed = 1/16 º

Goniometer arm length: 240 mm

Tube voltage and intensity: 45 kV, 40 mA

Temperature: 303 K

Scan range: 3.0 º to 75.0 º (2θ); scan step size: 0.017 º (2θ); counting time: 9972 s/step.

Crystallographic refinement data for ITQ-54

Chemical formula (per unit cell): $Si_{65.3}Ge_{62.7}O_{252}(OH)_8$

Chemical formula (Z = 128): $Si_{0.51}Ge_{0.49}O_{1.97}(OH)_{0.06}$

Density: 1.524 g/cm^3

Space group: *Immm* (No. 71)

a = 27.1592(20) Å, *b* = 25.8461(19) Å, *c* = 16.3764(11) Å

V = 11495.6(14) Å^3

2θ range = 3-75 º; stepsize (2θ) = 0.017 º

Peak range in FWHM = 25

Number of points = 4235; number of contributing reflections = 3989

Number of structural parameters = 90; number of profile parameters [(a)] = 12

Number of geometric restraints (dSi-O=1.61(1) Å) = 36

Number of geometric restraints (dSi-OH=1.65(1) Å) = 1

Number of geometric restraints (dGe-O=1.74(1) Å) = 36

Number of geometric restraints (dT-T=3.05(1) Å) = 21

R_{wp} = 0.095; R_{exp} = 0.035; R_B = 0.038; R_F = 0.041

[(a)] Including zero-shift and unit cell parameters.

Table S4. Atomic coordinates, thermal parameters and occupancy for calcined ITQ-54

Space group: *Immm* (#71); a = 27.1592(20) Å, b = 25.8461(19) Å, c = 16.3764(11) Å

T-site	Atom	x[(a)]	y[(a)]	z[(a)]	Occupancy	Uiso[(b)]	Multiplicity & Wyckoff
Si1	Si	0.44253(17)	0.2731(2)	0.0946(3)	0.322(12)	0.0178(8)	16o
Ge1	Ge	0.44253(17)	0.2731(2)	0.0946(3)	0.678(12)	0.0178(8)	16o
Si2	Si	0.2073(2)	0.1561(2)	0.2514(4)	0.435(18)	0.0178(8)	16o
Ge2	Ge	0.2073(2)	0.1561(2)	0.2514(4)	0.565(18)	0.0178(8)	16o
Si3	Si	0.2499(2)	0.2100(2)	0.0950(3)	0.327(15)	0.0178(8)	16o
Ge3	Ge	0.2499(2)	0.2100(2)	0.0950(3)	0.673(15)	0.0178(8)	16o
Si4	Si	0.05694(17)	0.1058(2)	0.4059(3)	0.535(14)	0.0178(8)	16o
Ge4	Ge	0.05694(17)	0.1058(2)	0.4059(3)	0.465(14)	0.0178(8)	16o
Si5	Si	0.1993(2)	0.3118(2)	0.1505(3)	0.389(16)	0.0178(8)	16o
Ge5	Ge	0.1993(2)	0.3118(2)	0.1505(3)	0.612(16)	0.0178(8)	16o
Si6	Si	0.1571(2)	0.06024(19)	0.3397(4)	0.764(14)	0.0178(8)	16o
Ge6	Ge	0.1571(2)	0.06024(19)	0.3397(4)	0.236(14)	0.0178(8)	16o
Si7	Si	0.3443(2)	0.2425(2)	0.1914(4)	0.492(17)	0.0178(8)	16o
Ge7	Ge	0.3443(2)	0.2425(2)	0.1914(4)	0.508(17)	0.0178(8)	16o
Si8	Si	0.2195(3)	0.0589(2)	½	1	0.0178(8)	8n
Ge8	Ge	0.2195(3)	0.0589(2)	½	0	0.0178(8)	8n
Si9	Si	0.1800(3)	0.3851(3)	0	0.62(2)	0.0178(8)	8n
Ge9	Ge	0.1800(3)	0.3851(3)	0	0.38(2)	0.0178(8)	8n
O1	O	0.4005(9)	0.2384(10)	0.149(3)	1	0.057(6)	16o
O2	O	0.251(3)	0.1837(9)	0	1	0.057(6)	8n
O3	O	0.2415(10)	0	½	1	0.057(6)	4f
O4	O	0.430(3)	0.2496(18)	0	1	0.057(6)	8n
O5	O	0	0.086(2)	0.381(4)	1	0.057(6)	8l
O6	O	0.1708(12)	0.3572(15)	0.0915(15)	1	0.057(6)	16o
O7	O	0.1961(15)	0.0751(13)	0.4124(17)	1	0.057(6)	16o
O8	O	0.0981(6)	0.0748(20)	0.347(2)	1	0.057(6)	16o
O9	O	0.1810(18)	0.0978(10)	0.2687(12)	1	0.057(6)	16o
O10	O	0.2183(18)	0.1649(13)	0.1504(7)	1	0.057(6)	16o
O11	O	0.3072(9)	0.2031(9)	0.137(3)	1	0.057(6)	16o
O12	O	0.2321(18)	0.2735(8)	0.0855(18)	1	0.057(6)	16o

O13	O	0.072(2)	0.087(2)	½	1	0.057(6)	8n
O14	O	½	0.2513(20)	0.117(4)	1	0.057(6)	8l
O15	O	0.2600(4)	0.1047(7)	½	1	0.057(6)	8n
O16	O	0.421(1)	0.3345(3)	0.111(3)	1	0.057(6)	16o
O17	O	0.2602(11)	0.1489(7)	0.303(3)	1	0.057(6)	16o
O18	O	0.155(3)	0	0.3029(17)	1	0.057(6)	8m
O19	O	0.1599(10)	0.294(2)	0.224(2)	1	0.057(6)	16o
OH20	OH	0.157(2)	0.4452(10)	0	1	0.09(5)	8n
O21	O	0.3291(16)	0.3055(6)	0.192(2)	1	0.057(6)	16o

Numbers in parentheses are the esd's in the units of the least significant digit given.

(a) Parameters without an esd were not refined. Coordinates equal to 0 or ½ are fixed by symmetry. (b) All the Si atoms have been refined with a common U_{iso} parameter; the same applies for all the O atoms.

Interatomic bond distances (Å)

T1-O4=1.70(2)	T1-O14=1.67(2)	T1-O1=1.70(3)	T1-O16=1.71(1)
T2-O17=1.68(4)	T2-O21=1.68(3)	T2-O9=1.69(3)	T2-O10=1.70(2)
T3-O2=1.70(1)	T3-O10=1.71(3)	T3-O11=1.71(3)	T3-O12=1.72(2)
T4-O13=1.67(2)	T4-O16=1.68(1)	T4-O5=1.68(2)	T4-O8=1.68(3)
T5-O19=1.68(3)	T5-O17=1.68(3)	T5-O12=1.70(3)	T5-O6=1.71(3)
T6-O7=1.64(3)	T6-O9=1.65(3)	T6-O8=1.65(2)	T6-O18=1.67(1)
T7-O21=1.68(2)	T7-O19=1.68(4)	T7-O1=1.68(3)	T7-O11=1.69(3)
T8-O15=1.62(2)	T8-O7=1.62(3)	T8-O7=1.62(3)	T8-O3=1.64(1)
T9-O15=1.65(1)	T9-OH20=1.67(3)	T9-O6=1.68(3)	T9-O6=1.68(3)

S6. Structure and topology analysis of ITQ-54

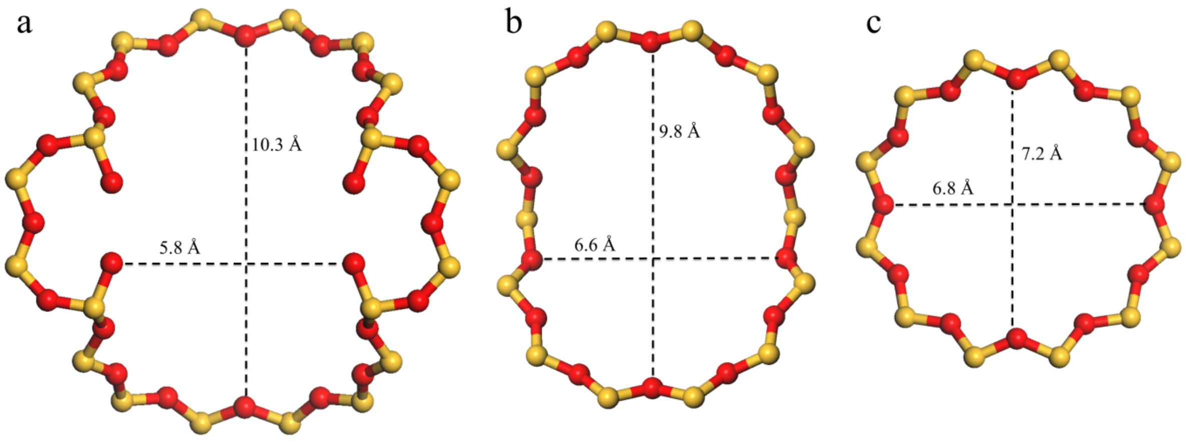

Figure S6. Maximum effective pore diameters of (a) 20-ring, (b) 14-ring and (c) 12-ring windows in ITQ-54, estimated assuming the van der Waals diameter of oxygen 2.7 Å. T atoms are yellow and O atoms in red.

Topology analysis was performed using TOPOS.[36,37] The visualisation of nets and tiling was generated using *3dt*.[37] There are two types of D4Rs [4^6]; D4R1 is generated from T2, T3, T5 and T7 and D4R2 from T1 and T4. The coordination sequences and vertex symbols are given in Table S5. The transitivity of the ITQ-54 framework based on the T-atoms is **9 20 20 10**.

Table S5. Coordination sequences and vertex symbols of ITQ-54

T-atom name	N1 to N10	Vertex Symbol
T1	4 9 16 24 35 50 69 96 124 146	4•5•4•6•4•14
T2	4 9 16 25 33 49 70 91 112 148	4•5•4•5•4•12
T3	4 9 15 23 36 53 70 86 111 139	4•5•4•6•4•12
T4	4 9 17 26 33 44 64 91 123 154	4•5•4•5•4•8
T5	4 8 15 22 33 53 70 91 109 138	4•5•4•5•4•14
T6	4 11 18 25 35 48 65 92 127 152	4•5•5•8•5•12
T7	4 9 15 23 36 51 72 89 118 142	4•5•4•6•4•14
T8	4 9 18 24 33 50 67 99 130 142	4•5•4•5•5•20(12)
T9	3 9 13 22 36 45 77 90 110 144	Rings coincide with circuits

Each center of the [$4^3 5^6 6^1$] CBU lies on the nodes of the four coordinated **crb** nets (Figure S7a). The channel system consists of two unique large cavities (Figure S7b). Each [$4^2 6^4$] connect to [$6^2 8^2$] along a-axis to form 12-ring and along b-axis to form 14-ring channels, respectively. Besides the 12-ring along a-axis, each [$6^2 8^2$] also connects to another [$6^2 8^2$] along c-axis to form 20-ring channels.

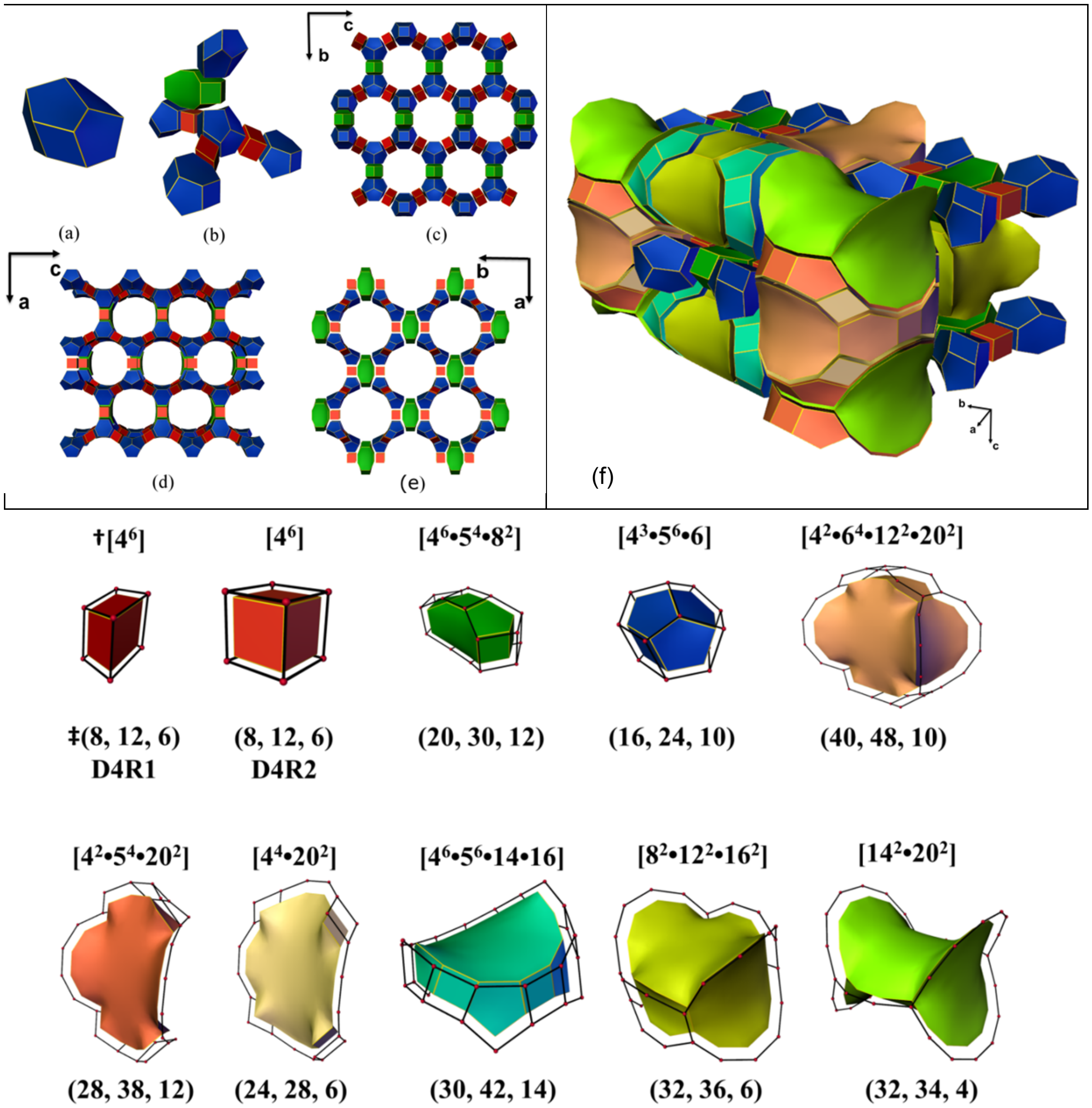

Figure S7. Construction of the structure of ITQ-54 using tiling. (a) The new [$4^35^66^1$] CBU. (b) The connectivity of the new CBUs: each CBU connects to four other CBUs via three [4^6] tiles (D4Rs, in red and orange) and one [$4^65^48^2$] (in green). (c-e) The structure of ITQ-54 viewed along the (c) *a*-axis, (d) *b*-axis and (e) *c*-axis showing the 12-, 14- and 20-ring channels, respectively. (f) Illustration of the channel system and cavities in ITQ-54 by tiles. There are three large tiles representing the cavities in ITQ-54; [$4^26^412^220^2$], [$8^212^216^2$] and [14^220^2]. Each [$4^26^412^220^2$] cavity connects the [$8^212^216^2$] cavities via their 12-ring windows along *a*, and the [14^220^2] cavities via the 20-ring windows of [4^420^2] and [$4^25^420^2$] as connectors along *c*. The [$8^212^216^2$] cavity connects the [14^220^2] cavities along *b*-axis via the bridging [$4^65^614^116^1$] tiles. Although the [$8^212^216^2$] cavities have 16-ring windows, the channels have 14-ring windows delimited by the [14^220^2] cavities. The ten different tiles in ITQ-54 are shown in the bottom. Detailed nets and *tiling construction of ITQ-54 are in *Immm*. †[Face symbol], ‡(V, E, F): number of vertices, number of edges, number of faces. *Tiling signature: **6[4^6] + 2[$4^65^614^116^1$] + [$4^65^48^2$] + 2[4^420^2] + 4[$4^35^66^1$] + 2 [$4^25^420^2$] + [$4^26^412^220^2$] + [$8^212^216^2$] + [14^220^2].**

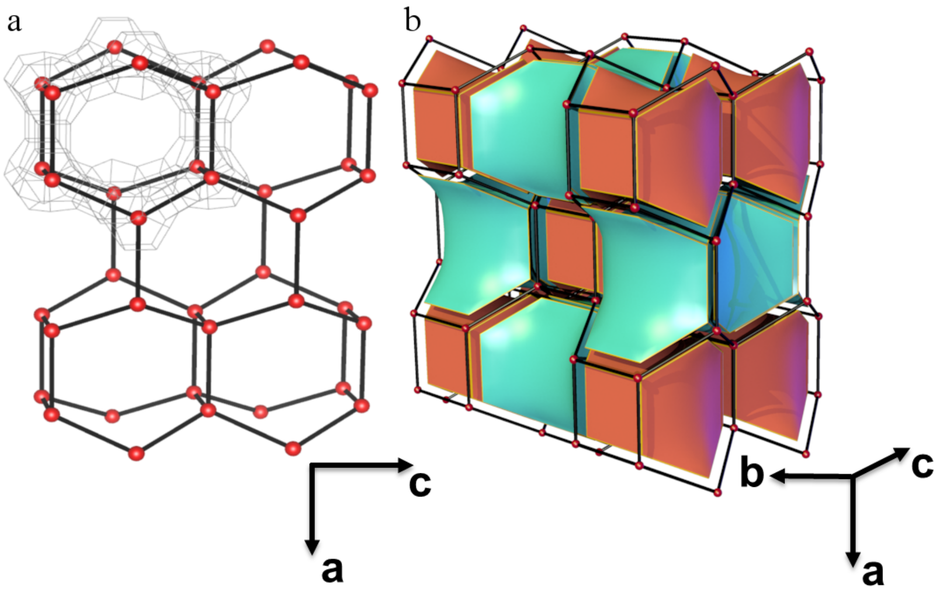

Figure S8. The underlying topology and tiling of ITQ-54 based on the [$4^3 5^6 6$] CBU. (a) The underlying topology is the **crb** net, the centers of the [$4^3 5^6 6$] CBUs fall on the nodes (in red) of the **crb** net. The T-T connections of ITQ-54 are shown in grey. (b) The channels of ITQ-54 represented by two types of tiles; a [$4^2 6^4$] cage (in brown) and a [$6^2 8^2$] cavity (in blue).

S7. Characterisation of OSDA

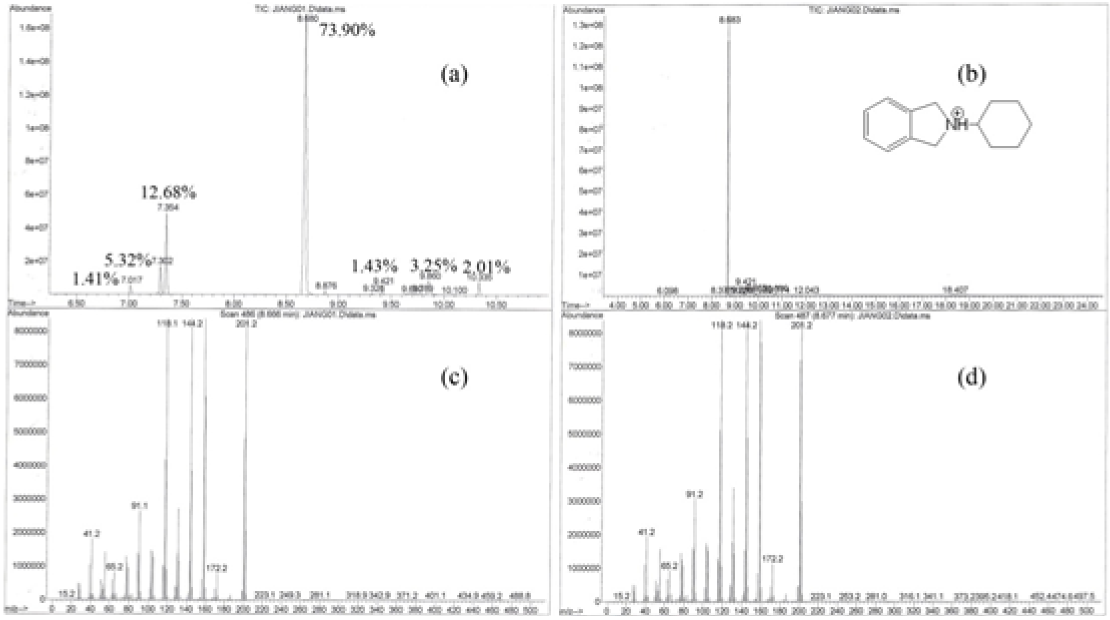

Figure S9. (a) The gas chromatography spectrum of guest molecules (cations) which decomposed in-situ from OSDA. The organic species can be estimated by integrated peak area. (b) The gas chromatography spectrum of synthetic protonated amine CHI. (c) Mass spectrum of main component of decomposed OSDA (peak at 8.68min). (d) Mass spectrum of synthetic protonated amine CHI.

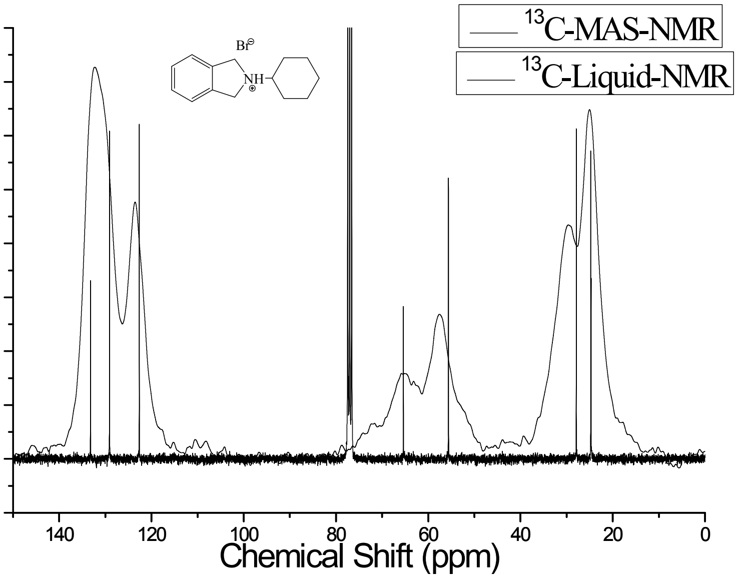

Figure S10. Comparison between solid state ^{13}C-MAS-NMR of zeolite sample and ^{13}C-Liquid-NMR of synthetic protonated amine CHI which was also detected as a main component of decomposed OSDA.

S8. Textural properties of ITQ-54

To study the textural properties of ITQ-54, N_2 and Ar adsorption measurements were performed on the calcined material. The t-plot method applied to the N_2 adsorption at 77 K reveals a micropore volume of 0.146 cm^3/g and a micropore area of 298 m^2/g. The total Brunauer-Emmet-Teller (BET) surface area is 333 m^2/g. The N_2 adsorption isotherm shows the characteristic shape of microporous materials (Figure S10a). Interestingly, Ar adsorption at 87 K exhibits an experimental pore distribution calculated by applying the Horvath–Kawazoe formalism, centered at 8.1 Å (Figure S 10b).

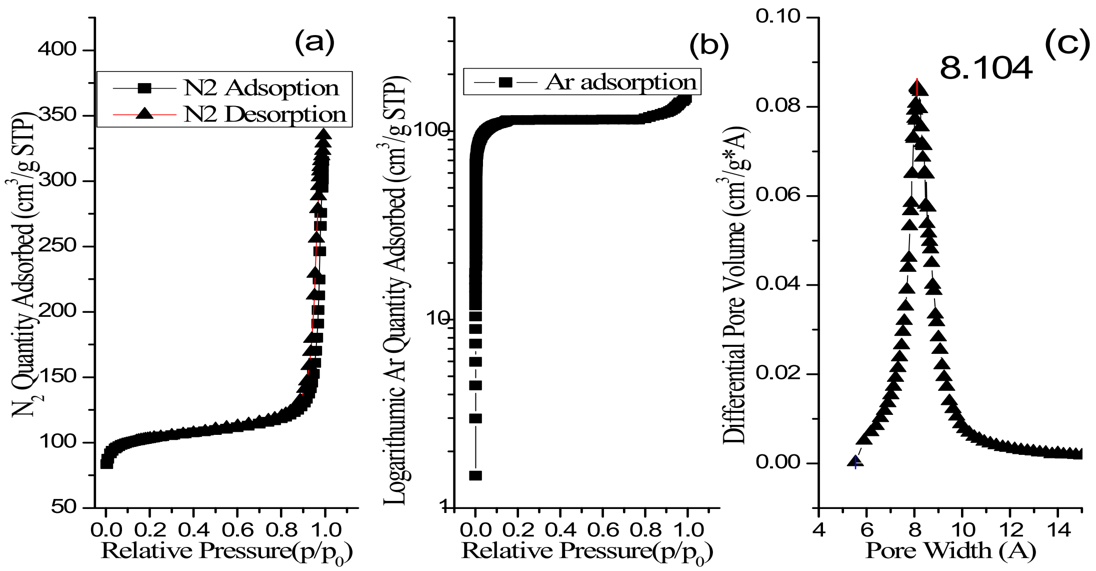

Figure S11. (a) N_2 adsorption and desorption isotherm. (b) Logarithmic isotherm of Argon adsorption (c) Horvath-Kawazoe Differential Pore Volume vs width distribution.

S9. ^{29}Si and ^{19}F Solid state NMR

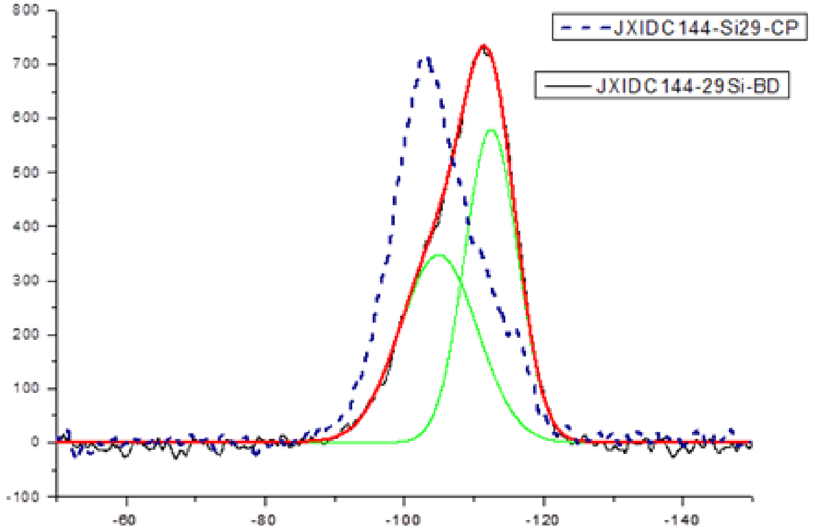

Figure S12. Comparison between ^{29}Si BD MAS-NMR and ^{1}H to ^{29}Si CPMAS-NMR shows that the Q^3 (Si-O-H) peak (-105ppm) has been increased in ^{1}H to ^{29}Si CP MAS-NMR spectrum. The red line corresponds to ^{29}Si BD MAS-NMR spectrum which was fitted as two peaks (shown as in green line). The dash line corresponds to ^{1}H to ^{29}Si CP MAS-NMR.

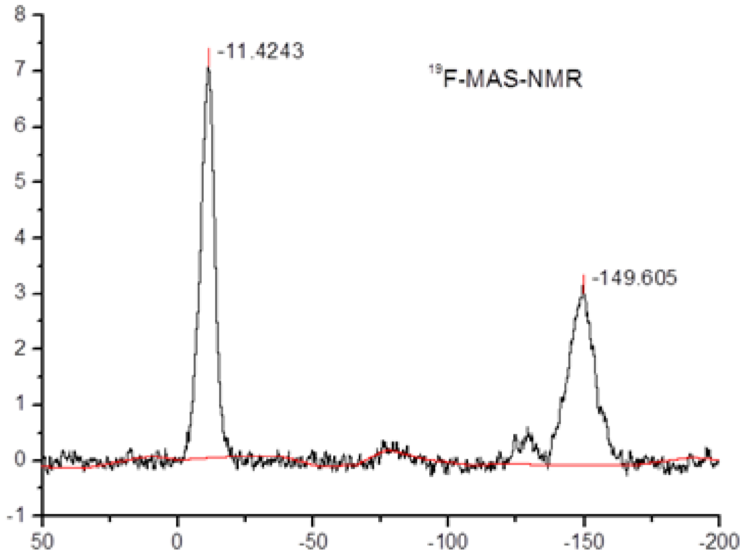

Figure S13. ^{19}F MAS-NMR spectrum of the as-made ITQ-54. The peak at -11.42 ppm corresponds to the F^- ions in the D4Rs.

S10. Thermogravimetrical analysis

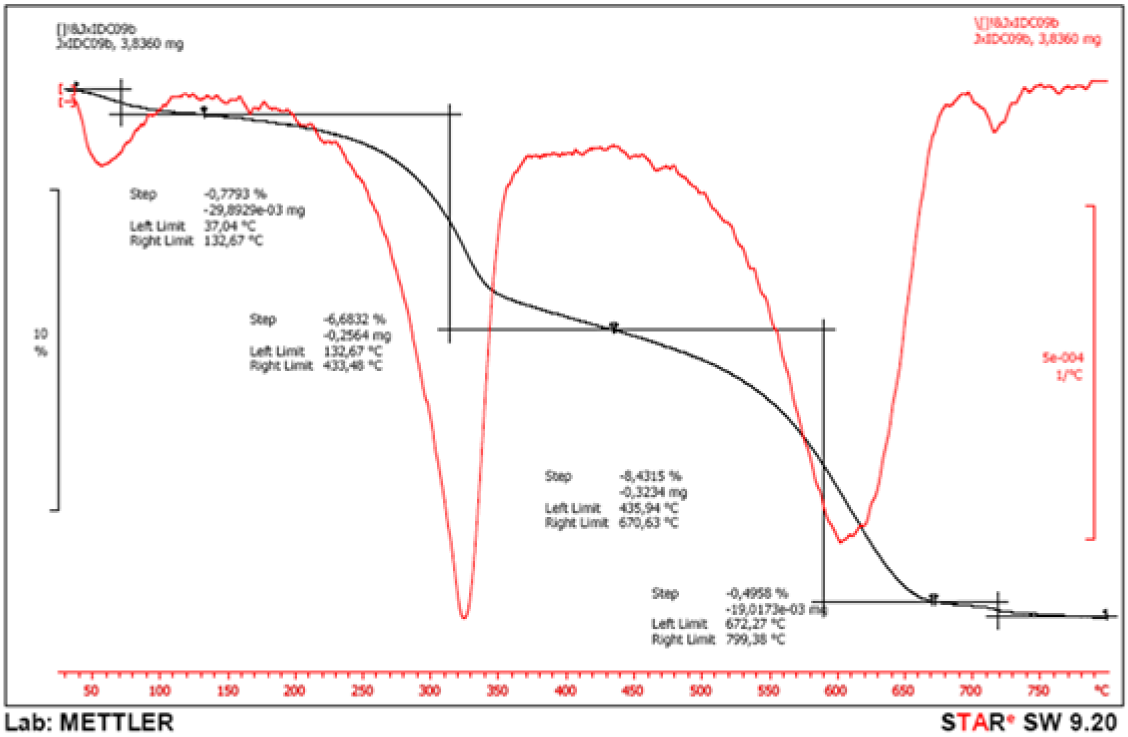

Figure S14. Thermogravimetrical analysis of as-made ITQ-54.

References

39. J. Jiang, J. L. Jorda, M. J. Diaz-Cabanas, J. Yu, and A. Corma, *Angew. Chem. Int. Ed.,* 2010, **49**, 4986 –4988.
40. J. Callahan, *J. Sediment. Res.,* 1987, **57**.
41. M. R. GREGORY, K. A. JOHNSTON, *N. Z. J. Geol. Geophys.,* 1987, **30**, 317–320.
42. M. A. Caffrey, S. P. Horn, *Palynology,* 2013, **37**, 143–150.
